# Aggregation of Albumins under Reductive Radical Stress

**DOI:** 10.3390/ijms25169009

**Published:** 2024-08-19

**Authors:** Karolina Radomska, Liwia Lebelt, Marian Wolszczak

**Affiliations:** 1 Institute of Applied Radiation Chemistry, Faculty of Chemistry, Lodz University of Technology, 93-590 Lodz, Poland; karolina.radomska@p.lodz.pl; 2 Centre of Papermaking and Printing, Lodz University of Technology, Wolczanska 221, 93-005 Lodz, Poland; 3 Bioorganic Chemistry Laboratory, Faculty of Pharmacy, Medical University of Lodz, Muszynskiego 1, 90-151 Lodz, Poland; liwia.lebelt@umed.lodz.pl

**Keywords:** aggregation, albumins, pulse radiolysis, reductive radical stress

## Abstract

The reactions of radicals with human serum albumin (HSA) under reductive stress conditions were studied using pulse radiolysis and photochemical methods. It was proved that irradiation of HSA solutions under reductive stress conditions results in the formation of stable protein aggregates. HSA aggregates induced by ionizing radiation are characterized by unique emission, different from the UV emission of non-irradiated solutions. The comparison of transient absorption spectra and the reactivity of hydrated electrons ($e_{aq}^{-}$) with amino acids or HSA suggests that electron attachment to disulfide bonds is responsible for the transient spectrum recorded in the case of albumin solutions. The reactions of $e_{aq}^{-}$ and ${CO}_{2}^{•-}$ with HSA lead to the formation of the same products. Recombination of sulfur-centered radicals plays a crucial role in the generation of HSA nanoparticles, which are stabilized by intermolecular disulfide bonds. The process of creating disulfide bridges under the influence of ionizing radiation is a promising method for the synthesis of biocompatible protein nanostructures for medical applications. Our Raman spectroscopy studies indicate strong modification of disulfide bonds and confirm the aggregation of albumins as well. Low-temperature measurements indicate the possibility of electron tunneling through the HSA protein structure to specific CyS-SCy bridges. The current study showed that the efficiency of HSA aggregation depends on two main factors: dose rate (number of pulses per unit time in the case of pulse radiolysis) and the temperature of the irradiated solution.

## 1. Introduction

Albumin nanostructures have many applications, including in medicine as drug carriers, in anticancer therapy and in cosmetology [1,2]. They are used, among other reasons, for the controlled release of active molecules and substances in organisms, including drugs, genes, peptides, vaccines and antibodies. An interesting application of nanotechnology in medicine is nanoparticles that can replace viruses used in gene therapy [3]. Nanoscale protein complexes can transport therapeutic DNA fragments inside cells in a safe way for the organism. Scientists from the Institute of Biotechnology and Biomedicine at the Universitat Autònoma de Barcelona managed to obtain artificial viruses in the form of protein complexes that self-form into nanoparticles around DNA fragments and are transported inside cells [4]. Another interesting application of protein nanostructures is nano-eye-drops. The technology was developed by researchers at Bar-Ilan University’s Institute of Nanotechnology and Advanced Materials [5]. They have created non-toxic eye-drops with nanoproteins that can correct vision defects such as farsightedness and myopia.

Drug delivery systems include various types of systems, including nanostructures, micelles, protein–drug systems and liposome–drug systems. The unique properties of human albumin (it is an endogenous protein, non-toxic, non-antigenic and stable in a wide pH range) make it suitable for use as a drug carrier. Moreover, human serum albumin is a promising drug delivery agent due to its biocompatibility, biodegradability, lack of immunogenicity and toxicity [6,7]. There are two strategies for using human albumin as a drug carrier. The first one involves the production of conjugates, i.e., complexes containing a single chain of HSA to which the drug is chemically bound [8]. The second method is to produce protein nanoparticles in which the drug is encapsulated. These drugs include peptides, nucleic acids, antibodies, cytokines and enzymes. Encapsulation of the drug into the protein nanostructure without covalent bond formation seems to be a better method for drugs that, e.g., are poorly soluble in water. Protein nanostructures may be effective in the treatment of many diseases, including cancer therapy, since nanoparticles smaller than 200 nm accumulate in tumours due to their enhanced permeation and retention (EPR) effect [9]. This type of nanoparticle displays a long half-life (~19 days) [10]. Another advantage of HSA nanostructures is their ability to release drugs “on demand” depending on the microenvironment of the lesion [7]. Due to this property of albumin nanostructures, better drug pharmacokinetics and drug targeting are achieved.

Various technologies are used to produce protein nanoparticles, such as coacervation, emulsion and desolvation methods [11,12]. Using the desolvation technique, Kimura et al. have prepared doxorubicin–HSA nanoparticles (with a particle size ~108 nm) using ethanol as a desolvating agent [9]. The addition of alcohol removes water molecules from the nearest solvation layer right at the protein surface, which causes the formation of polypeptide bonds between albumin molecules.

To optimize the preparation process of nanoparticles of albumin, various process parameters were studied: albumin and glutaraldehyde concentration, pH value of an albumin solution and rate of ethanol addition [8]. The measurement results indicate, among other things, that the pH of the HSA solution is an important parameter for effective particle size control in the desolvation method (smaller nanoparticles are formed in a solution with a higher pH).

The literature also describes the spray-drying method [13], Nab technology [14] and the protein self-assembly method [15]. Chemical crosslinking agents such as glutaraldehyde are used to stabilize albumin nanoparticles, but they also cause chemical modification of the protein. Furthermore, it is necessary to remove excess aldehyde after the reaction, which prevents the use of crosslinking agents in in vivo applications. For this reason, we became interested in using ionizing radiation to obtain protein nanostructures. The advantages of the ionizing radiation are related to the absence of toxic compounds and the possibility of nanostructure formation with simultaneous in situ sterilization in the final package.

Protein modifications associated with one-electron oxidation have been described in detail in the literature [16,17,18,19,20,21]. In contrast to oxidative stress, the process of albumin reduction is not so well characterized [22,23,24]. It has been postulated that carbonyl groups in HSA, especially when exposed to the aqueous phase, are the main targets of attack by hydrated electrons [25]. HSA contains 35 Cys (cysteine) residues that form 17 disulfide bridges (CyS-SCy) and 1 unpaired cysteine (Cys34) with unique thiol functionality. Salzano et. al. described the characteristics of HSA damage by reactive reducing species ($e_{aq}^{-}$, H^•^) [23]. Raman spectroscopy and mass spectrometry experiments have shown that amino acids containing sulfur in their structure undergo modifications (Met and Cys residues: Cys200-Cys246, Cys392–438, Cys514-Cys559). Reactions of these amino acids with a hydrated electron or hydrogen atom lead to the generation of thiyl radicals. As a result of the reaction of the hydrated electron with CyS-SCy residues in various HSA molecules, the following process takes place:

CyS−SCy+eaq−→CyS−SCy−•↔CyS•+CyS−

Intermolecular recombination of CyS^•^ radicals leads to aggregation of HSA as a result of the formation of S-S bridges for adjacent reduced albumin molecules.

The main aim of our research is to study the reduction process of proteins. Irradiation of aqueous solutions containing *t-*BuOH has been used for generating reducing species, namely, $e_{aq}^{-}$ and H^•^. Another reducing radical used in our measurements was the ${CO}_{2}^{•-}$ radical anion, which differs from the above-mentioned radicals in terms of redox potential, size and diffusion coefficient. The process of reducing proteins related to the transfer of one electron was studied by pulse radiolysis (at room temperature and 77 K), photochemical methods, Raman spectroscopy and HPLC measurements. It was proved that irradiation of HSA and BSA solutions under reductive stress conditions results in the formation of stable low- and high-molecular-weight protein aggregates.

## 2. Results

### 2.1. Pulse Radiolysis of Albumin Solutions

**Figure 1:**
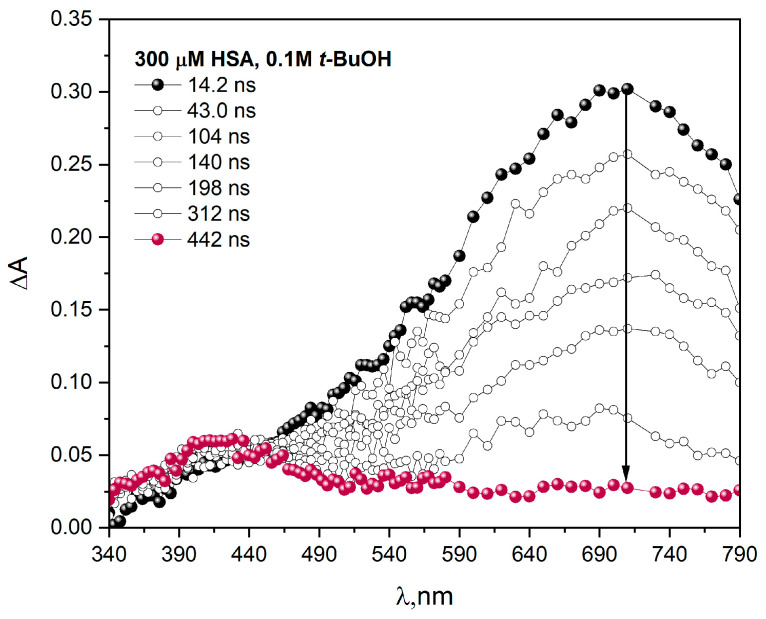
Transient absorption spectra were recorded at various times after 17 ns pulse irradiation with a dose of 54 Gy at 295 K of N_2_-saturated buffer solution, containing 300 μM HSA and *t-*BuOH (0.1 M). Phosphate buffer, pH 7.2.

The main purpose of our research was to study the reactions induced by ionizing radiation in protein aqueous solutions under reductive radical stress. To study the reaction of hydrated electrons with human serum albumin, we applied pulse radiolysis. Figure 1 shows transient absorption spectra recorded at various times after the electron pulse irradiation of N_2_-saturated aqueous buffer solution containing 300 µM HSA. ΔA represents the difference in absorbance compared to the non-irradiated sample.

A broad absorption band of $e_{aq}^{-}$ with a maximum at ~720 nm was observed immediately after irradiation of the buffer solution of HSA (300 μM) containing *t-*BuOH (0.1 M). This band disappeared within a microsecond time scale [1]. New absorption in the visible region with a maximum near 420 nm is due to the reduction product of HSA. The main process of reaction of a hydrated electron with HSA is the formation of the disulfide radical anion (CyS-SCy^•−^). Referring to our previous work (and references therein), we have proven that disulfide bridges are the most reactive residue in the capture of hydrated electrons by HSA [1]. We conducted pulse radiolysis experiments separately for aqueous solutions containing amino acids, including cysteine or cystine, and for the solution of HSA. The analysis of the shape of the transient absorption spectra and the reactivity of $e_{aq}^{-}$ with HSA and amino acids suggests that electron attachment to disulfide bonds (CyS-SCy^•−^) is responsible for the spectrum with a maximum at 420 nm that was recorded in the case of pulse radiolysis of the protein solution [1].

**Figure 2:**
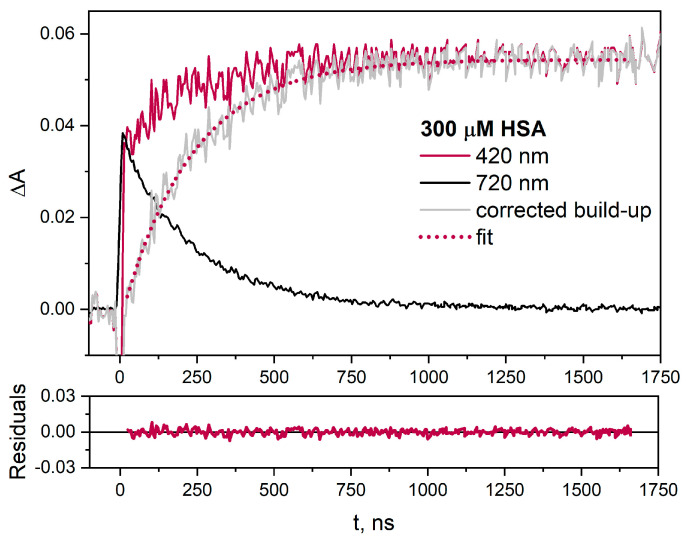
Time profiles of the absorbance were recorded in N_2_−saturated buffer solution containing HSA (300 µM) and *t-*BuOH (0.1 M) at λ =420 nm (pink line), obtained for the irradiation dose 40 Gy. The normalized decay of $e_{aq}^{-}$ was recorded at λ = 720 nm (black line) and the corrected growth at λ = 420 nm (grey line) using the described procedure.

The kinetic absorption pattern at 420 nm is complex. Due to the strong overlapping of the absorption bands of the hydrated electrons (~420 nm) and CyS-SCy^•−^ radicals (λ_max_ = 420 nm), the formation of disulfide radical anions cannot be directly used to calculate the rate constant of $e_{aq}^{-}$ scavenging by HSA. For this reason, we corrected the rate constant of the CyS-SCy^•−^ radical formation (a decay at λ = 420 nm), taking into account the decay of the $e_{aq}^{-}$ at 720 nm where the reduction product of HSA does not absorb light (Figure 2). The normalized decay of the hydrated electron was subtracted from the absorption trace recorded at 420 nm. After correction, it can be concluded that the decay of $e_{aq}^{-}$ and formation of CyS-SCy^•−^ radicals evolve at the same rate (k_decay_ = (1/232 ns) = 1.43 · 10^6^ s^−1^; k_formation_ = (1/228 ns) = 1.46 · 10^6^ s^−1^).

We also observed that the absorbance of hydrated electrons (λ_max_~720 nm) produced in the albumin solution, even at high concentrations (above 300 μM), was only about 2% lower compared to the neat buffer solution for the same dose. The difference in the initial hydrated electron absorbance value can be attributed to dry electron capture by HSA. In our opinion, the percentage of dry electrons scavenged by proteins in pulse radiolysis is insignificant and may be omitted in the analysis of the HSA reduction process. In the case of other proteins, the contribution of the dry electron to the albumin scavenging process may be different [26]. In the case of pulse radiolysis of protein solutions isolated from hen egg whites, the contribution of dry electrons in the scavenging was much higher than in the case of the studied albumins and amounted to 23%.

**Figure 3:**
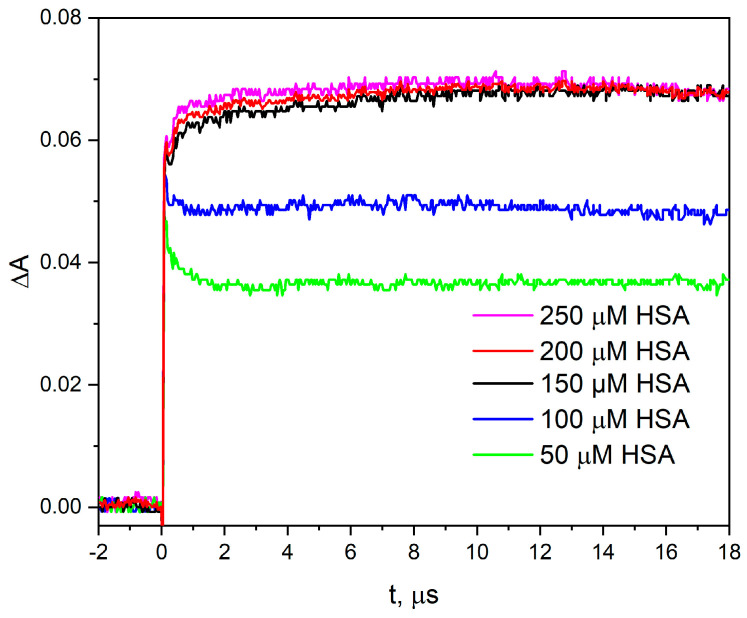
Time profiles of the absorbance were recorded at 420 nm after 17 ns pulse irradiation with a dose of 54 Gy of the N_2_−saturated aqueous solution containing HSA (50–250 μM) and *t-*BuOH (0.1 M). The molar absorption coefficient ε of reduced HSA (ε for CyS−SCy^•−^) is 9·10^3^ dm^3^ mol^−1^ s^−1^.

We estimated that at pH 7.2, about 50% of all electrons produced in pulse radiolysis (15.0 µM) contributed to the formation of 7.7 µM CyS-SCy^•−^ radicals (Figure 3). The reduction process was monitored at 420 nm at the maximum of the absorbance band of the product of one electron-reduced HSA. This strongly indicates that not all electrons are attached to the disulfide residue. The remaining 50% of electrons reacted with the residues of the HSA polypeptide chain, and the primary capture products were not visible in the pulsed radiolysis measurements. It has been proven that these reactions of the hydrated electron with HSA mainly lead to the modification of several specific methionine residues [24].

**Figure 4:**
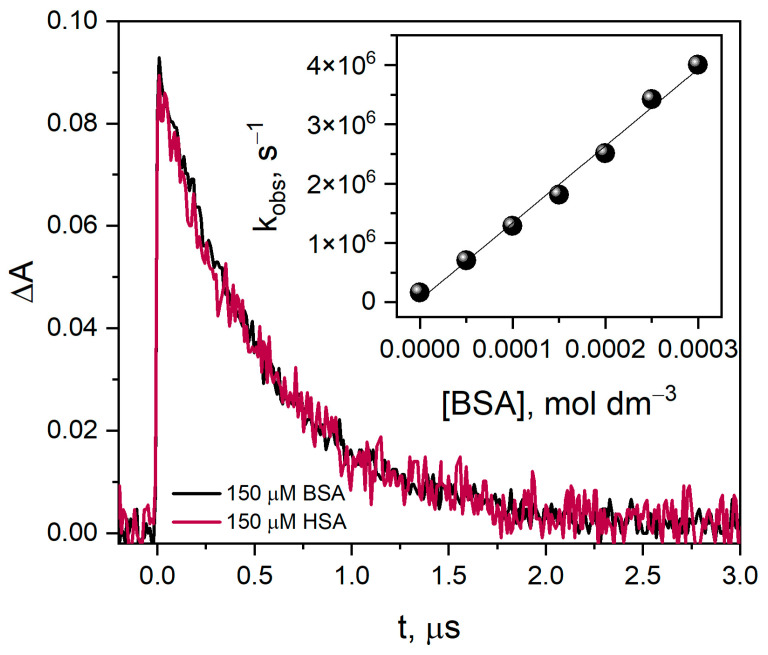
Time profiles of the absorbance were recorded at 720 nm after 7 ns pulse irradiation with a dose of 13 Gy of the vacuum-deaerated aqueous solution containing HSA or BSA (150 µM) and *t-*BuOH (0.1 M) at 295 K. Insert. The rate constant k_obs_ for the reaction of a hydrated electron with BSA as a function of protein concentration (0–300 µM). Phosphate buffer, pH 7.2.

Based on the literature data [24,27] and our measurements, it was proved that the modifications ascertained in the protein structure were highly dependent on the system under investigation and the experimental conditions used during sample irradiation. To accurately determine the yield of CyS-SCy^•−^ radicals, the concentration of scavengers should exceed the hydrated electron concentration. Figure 3 shows the kinetics of the formation of the electron–protein reaction product for several HSA concentrations. An electron beam was used for the measurements, which produced approximately 15 µM hydrated electrons in each of the tested solutions. For HSA concentrations below 150 µM, not all electrons were captured by the protein. Moreover, when analyzing the rate constant of hydrated electron decay for various proteins, the measurements must be carried out with the same buffer solution and the ratio of protein concentration to $e_{aq}^{-}$ concentration, preferably in the same measurement series. In our opinion, a protein concentration ten times higher than the concentration of the hydrated electron produced during radiolysis measurement should be used. According to the mentioned rules, it can be concluded that the decay of hydrated electron in HSA or BSA solution evolves at the same rate (Figure 4).

In the buffer solution, the reaction of the hydrated electron with HSA or BSA is diffusion-controlled. The rate of the reaction was determined from the hydrated electron decay in the vacuum-deaerated aqueous solution of BSA containing 0.1 M *t-*BuOH (absorbance at 720 nm, at room temperature) for various protein concentrations. The decay curves, absorbance vs. time, were fitted by the pseudo-first-order kinetic equation. The dependence of the rate constant for the decay of the absorption band of hydrated electrons in the reaction with BSA is linear over the entire range of protein concentrations (Inset Figure 4). From the slope of the line k_obs_ vs. BSA concentration, the second-order rate constant for scavenging was calculated as 1.3 × 10^10^ dm^3^ mol^−1^ s^−1^. This value is in good agreement with the value we previously obtained for the rate constant of the reaction $e_{aq}^{-}$ with HSA: 1.1 × 10^10^ dm^3^ mol^−1^ s^−1^ [1,28].

**Figure 5:**
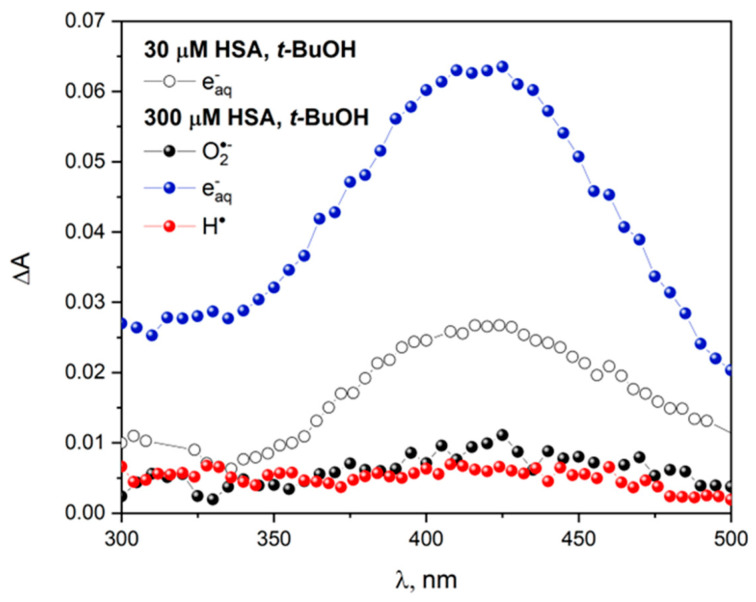
Transient absorption spectra of N_2_-saturated buffer solution containing 30 (○) or 300 (●) μM HSA and 0.1 M* t*-BuOH, obtained for irradiation dose 48 Gy; transient absorption spectra of O_2_-saturated buffer solution containing 300 μM HSA and 0.1 M* t*-BuOH, obtained for irradiation dose 48 Gy (●); transient absorption spectra of N_2_O-saturated buffer solution containing 300 μM HSA and 0.1 M* t*-BuOH, recorded for irradiation dose 48 Gy (●).

The reaction of radicals with albumins takes place in the physiological environment of a living cell. Free radicals are involved in many biological processes, including apoptosis or aging. For this reason, measurements under aerobic conditions were carried out to study the effect of oxygen on the HSA reduction process. It is well known that the energy barrier for the diffusion of oxygen molecules through the albumin structure is much higher than the analogous barrier in water. Similarly, the diffusion of reducing agents ($e_{aq}^{-}$, H^•^, ${CO}_{2}^{•-}$) in the internal albumin matrix is also inhibited. Figure 5 shows the transient absorption spectra recorded after the electron pulse irradiation of N_2_- or O_2_-saturated buffer solutions containing human serum albumin (30 or 300 μM) and *t-*BuOH (0.1 M). The oxygen concentration in an O_2_-saturated solution at room temperature is about 1.25 mM. The superoxide anion radical is formed as a result of one-electron reduction of oxygen by the hydrated electron. The value of absorption of the transient recorded during pulse radiolysis of 30 µM HSA is about 0.025 (λ_max_ = 420 nm) for a 40 Gy dose in the case of reaction with hydrated electrons (Figure 5). For a concentrated solution of albumin (300 μM HSA), this value increases almost 2.5 times for the same dose. Saturation of the albumin solution with oxygen does not lead to the formation of the HSA reduction product. This is probably due to the low value of the reduction potential of superoxide anion radical $O_{2}^{•-}$ (E^0^ = −0.33 V) compared to $e_{aq}^{-}$ (E^0^ = −2.9 V).

The reaction of the hydrogen atom with HSA leads to the formation of very small amounts of CyS-SCy^•−^ based on the intensity of the band with a maximum of 420 nm (Figure 5). In steady-state measurements of HSA solutions irradiated with the same dose in the case of the reaction of $O_{2}^{•-}$ or H^•^ with albumin, we recorded identical absorption spectra. It should be emphasized that in the case of a solution containing HSA and saturated with oxygen, after irradiation, the radical anion $O_{2}^{•-}$ is formed as well as small numbers of hydrogen atoms.

**Figure 6:**
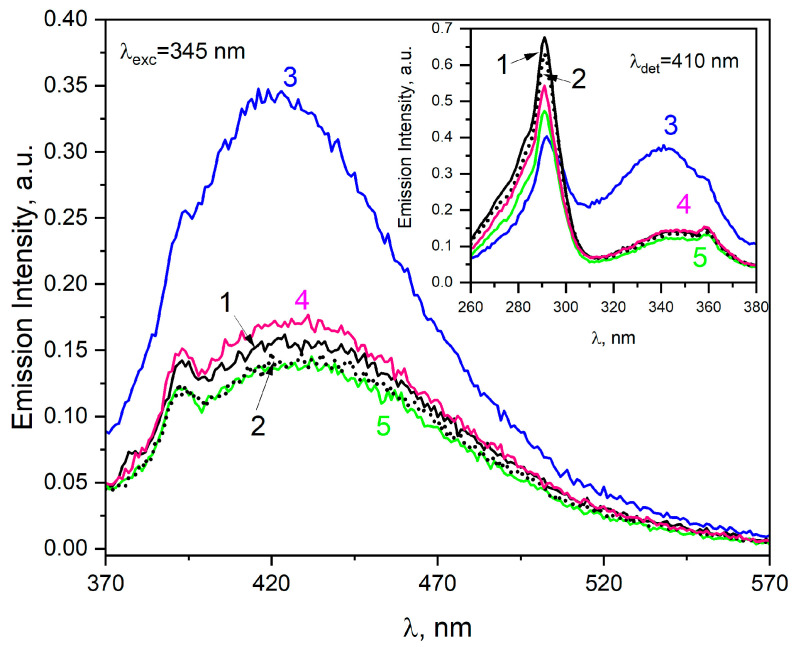
Emission spectra of the neat HSA solutions (30 μM, line 1) or HSA (30 μM) solution containing HCOONa (0.1 M, line 2). The emission spectrum of the vacuum-deaerated HSA solution (30 μM) containing *t-*BuOH (0.1 M), recorded after irradiation with dose 3500 Gy (line 3). The emission spectrum of the HSA solution (30 μM) saturated with oxygen-containing *t-*BuOH (0.1 M) was recorded after irradiation with a dose of 3500 Gy (line 4). The emission spectrum of the HSA solutions (30 μM) saturated with O_2_ containing HCOONa (0.1 M) and *t-*BuOH (0.1 M), recorded after irradiation with dose 3500 Gy, overlaps with spectrum before irradiation (line 5). The excitation wavelength was 345 nm. Insert. Emission excitation spectra of the above-mentioned HSA solutions. The emission wavelength was 410 nm.

Additionally, it was found that neither reagent ($O_{2}^{•-}$ or H^•^) led to the formation of HSA aggregates. This was evidenced by the absence of an emission spectrum typical of protein aggregates (Figure 6). Before irradiation, the emission spectrum of HSA showed one band with a maximum at 430 nm (λ_exc_ = 345 nm). After irradiation of the solution containing HSA (30 µM) and *t-*BuOH (0.1 M), the formation of a fluorescent product with maximum emission at 420 nm was observed (λ_exc_ = 345 nm). Saturation of the same solution with oxygen before radiation did not lead to emission centered at 420 nm (Figure 6). The emission spectrum of HSA recorded after irradiation of an O_2_-saturated solution containing *t-*BuOH is almost identical to that before irradiation. We recorded identical spectra after the reaction of the hydrogen atom with HSA and, in order not to complicate Figure 6, we omitted them. Therefore, an important conclusion can be drawn regarding the process of obtaining albumin nanostructures by irradiation, namely, the need to remove oxygen from the HSA or BSA solution before irradiation. Under the influence of air, radiolysis leads to a reduction in the effectiveness of the aggregation process and, even more unfavourably, to the scission of peptide chains. Most likely, $O_{2}^{•-}$ and H^•^ attack other -S-S- moieties within HSA than the hydrated electron. These initially reduced disulfide bridges separate and are reconnected, but intramolecularly, this leads to modification of the albumin without aggregation. In the case of albumin reduction by hydrated electrons, selected cystine groups are modified so that the best possible conditions for intermolecular -S-S- exchange are met. In the case of an O_2_-saturated solution containing HSA, t-BuOH (to remove hydroxyl radicals) and HCOONa, no new emission was observed after irradiation. This means that in a sample saturated with oxygen, the generated $e_{aq}^{-}$ reacts mainly with O_2_.

The rate constant for the reaction of the hydrated electron with HSA in buffer solution has been measured for the temperature range 278–318 K. The rate constant at various temperatures was determined based on the decay of $e_{aq}^{-}$ absorbance at 720 nm.

**Figure 7:**
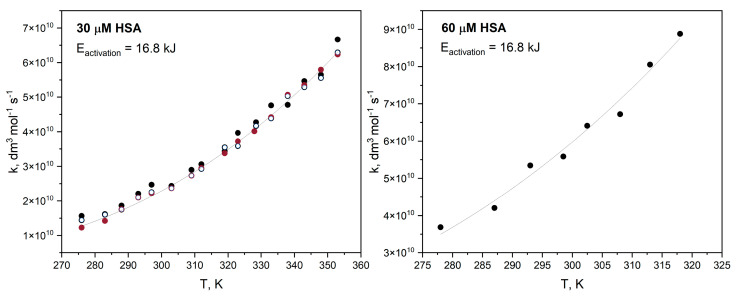
Rate constant for decay of hydrated electrons with HSA (60 µM) as a function of temperature (K). The energy of activation calculated from the Arrhenius equation is equal to ~16.8 kJ mol^−1^.

The Arrhenius expression describes the observed rate constant of electron scavenging by has relatively well. Nonlinear regression was conducted using OriginPro 2022 software to calculate the activation energy. For many years, the paradigm of radiation chemistry was that the activation energies for hydrated electron reactions are equal to the self-diffusion of water and tend to be in the 12–15 kJ mol^−1^ range. Most of the scavenging reactions of a hydrated electron are diffusion-controlled reactions and, as for HSA (60 µM), the energy of activation oscillates around the value 16.8 ± 1.0 kJ mol^−1^, as calculated by us (Figure 7). We performed the same measurements for a neat HSA solution with a greater concentration than 60 μM. Regardless of the protein concentration, we always obtained the same activation energy value. This result suggests that the thermodynamics of the reduction of amino acid residues in HSA is determined by the diffusion of the hydrated electron through bulk water to the HSA surface.

**Figure 8:**
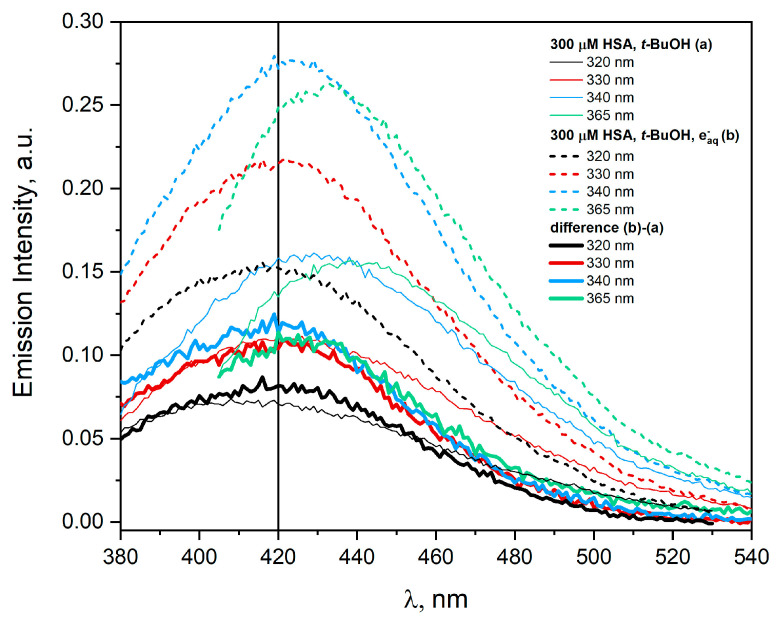
Emission spectra of the N_2_-saturated HSA solution (300 μM) containing *t-*BuOH (0.1 M), recorded before (a, thin solid lines) and after irradiation with a dose 25.2 kGy (b, dashed lines). After subtracting emission spectra (b) from (a), graphs with a maximum centered at 420 nm were obtained. The excitation wavelengths are given in the figure.

It is known from our experiments and from the literature that the reaction of HSA with a hydrated electron leads mainly to the reduction of the cystine residue [1,22,23,24,27]. Figure 8 shows the emission spectra of the HSA solution (300 µM) containing 0.1 M *t-*BuOH recorded before (a) and after (b) pulse radiolysis. The solution was saturated with N_2_ and irradiated with a dose of 25.2 kGy.

Spontaneous aggregation occurs in albumin solutions, and light-excited aggregates exhibit visible emission spectra before irradiation. The new fluorescence is associated with electron delocalization through the amino acid network of neighbouring HSA molecules and is not dependent on the presence of aromatic amino acids in albumin. In addition to hydrogen bonds that stabilize such aggregates, electrostatic forces, van der Waals forces and hydrophobic interactions are most likely also involved in the formation of new chromophore groups [17]. Emission spectra of spontaneous (non-covalent) HSA aggregates can be easily recorded for concentrations as low as 30 µM.

As a result of the reaction of a hydrated electron with HSA, aggregates of albumin are formed, which are characterized by emission centered at about 420 nm after excitation of the irradiated solution with 320 nm light. Aggregates are formed as a result of the intermolecular recombination reaction of disulfide radicals. The excitation of HSA solutions with light above 330 nm leads to the bathochromic shift of the emission band with a maximum at 420 nm. Subtracting the HSA emission spectra before and after irradiation shows that as a result of the reaction of a hydrated electron with albumin, one type of aggregate stabilized by covalent S-S bridges is formed (after subtracting, we observed emission centered near 420 nm for all excitation wavelengths). Although fluorescence (emission) is not an additive quantity, a procedure of subtracting fluorescence data from the sample background (in our case, the native fluorescence of concentrated albumin solutions) is often used [29].

The size of generated aggregates of albumin was monitored by dynamic light scattering (DLS). Analysis of DLS measurements after irradiation of N_2_-saturated solution containing HSA (300 μM) confirmed the formation of protein aggregates. Size measurements were made with a buffer solution of HSA (300 μM) and *t*-BuOH (0.1 M) before and after irradiation. HSA solutions (300 µM) were γ-irradiated with doses from 0 to 90 kGy. In the native solution, the mean size of albumin particles is about 7 nm. The average size of the HSA aggregates after irradiation was in the range of 17–38 nm. The conducted experiment confirmed that the size of HSA aggregates increases with the increase in the radiation dose (the size of protein aggregates depends linearly on the radiation dose). Using ionizing radiation, the formation of HSA nanostructures allows for control of aggregate size based on radiation dose and protein concentration.

The impact of radiation type (electron beam or gamma radiation) on the aggregation process of HSA was investigated. Emission excitation spectra of irradiated (electron beam or gamma radiation at 50 and 90 kGy, respectively) solutions containing HSA (420 μM) and *t*-BuOH (0.1 M) were recorded for different emission wavelengths (Figure S1 in Supplementary Materials). Spectroscopic measurements suggest the presence of two types of aggregates. Our previous spectroscopic measurements have shown that HSA undergoes reversible self-aggregation through protein–protein interactions [17]. This is probably a consequence of the formation of the hydrogen bond network and/or π–π interactions (van der Waals force) between HSA molecules. Disulfide bridges produced by the intermolecular recombination of CyS-SCy^•−^ radicals as a result of radiolysis of an aqueous protein solution are the main cause of HSA aggregation through crosslinking. The formation of aggregates stabilized by hydrogen bonds is preferable when the solution is irradiated by γ-radiation. The emission excitation spectra show two bands with a maximum at 364 and 383–396 nm (γ-radiation) or 382 nm and 382–396 nm (electron pulse). The intensity of emission of the red-shifted bands depends on the type of radiation used; the emission intensity is 1.5 times lower in the solution irradiated with an electron beam.

**Figure 9:**
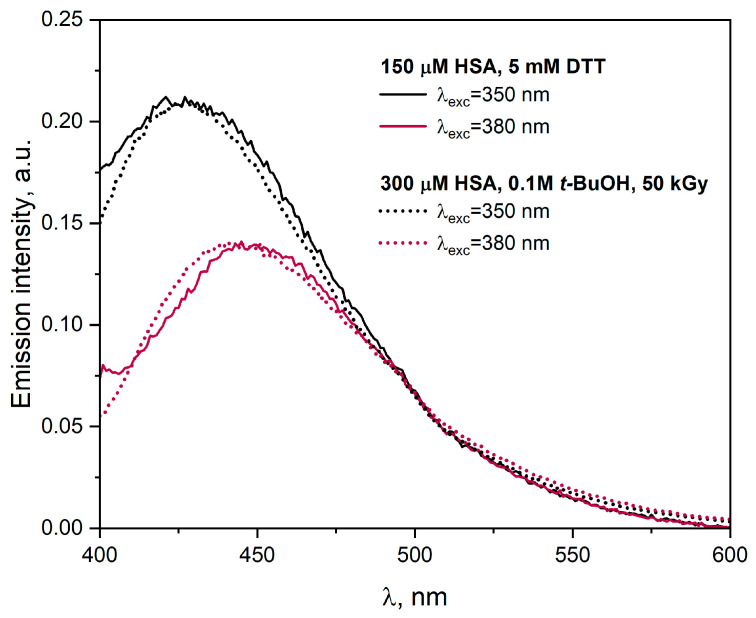
Emission spectra of the HSA solution (150 μM) and DTT (5 mM). Emission spectra of the N_2_-saturated HSA solutions (300 μM) containing 0.1 M *t-*BuOH recorded after γ-irradiation (50 kGy). The excitation wavelengths are given in the figure. Spectra were normalized to the same maximum intensity.

In our further work, we compared the process of HSA reduction by chemical and radiation methods. Dithiothreitol (DTT) was used as a chemical reducing agent for disulfide bonds [30]. The redox potential of DTT is −0.33 V at pH 7 [31]. Figure 9 shows the emission spectra of the HSA solution (150 or 300 µM) containing DTT (5 mM) or *t-*BuOH (0.1 M). The recommended DTT concentration for protein reduction is 1–10 mM [31]. The albumin solution with the addition of *t-*BuOH was deaerated and irradiated with a dose of 50 kGy. In the reaction with DTT, mainly S-S bonds within HSA, which are exposed to an aqueous solution, were reduced. Analysis of the HSA reduction process using chemical and radiation methods allowed us to confirm that -S-S- (CyS-SCy) bonds play the main role in both cases. The reaction of DTT or $e_{aq}^{-}$ with HSA led to the formation of similar products. We observed new emissions in the same spectral range in cases for $e_{aq}^{-}$ and DTT after the excitation of albumin solutions with 350 or 380 nm light (Figure 9). Recombination of the CyS-SCy^•−^ radicals led to the formation of protein aggregates stabilized by intermolecular disulfide bonds. However, considering the intensity of the HSA emission bands, a better method for obtaining protein aggregates is the radiation technique. For example, the intensity of the HSA emission band after excitation of the solution with 350 nm light was 3.5 times higher for $e_{aq}^{-}$ as a protein reductant than for DTT. The radiation method does not require the use of chemical crosslinkers, and it is also possible to simultaneously synthesize protein nanostructures and sterilize the final protein product. Moreover, as we mentioned above, by using different radiation doses, the size of the generated HSA aggregates can be controlled.

**Figure 10:**
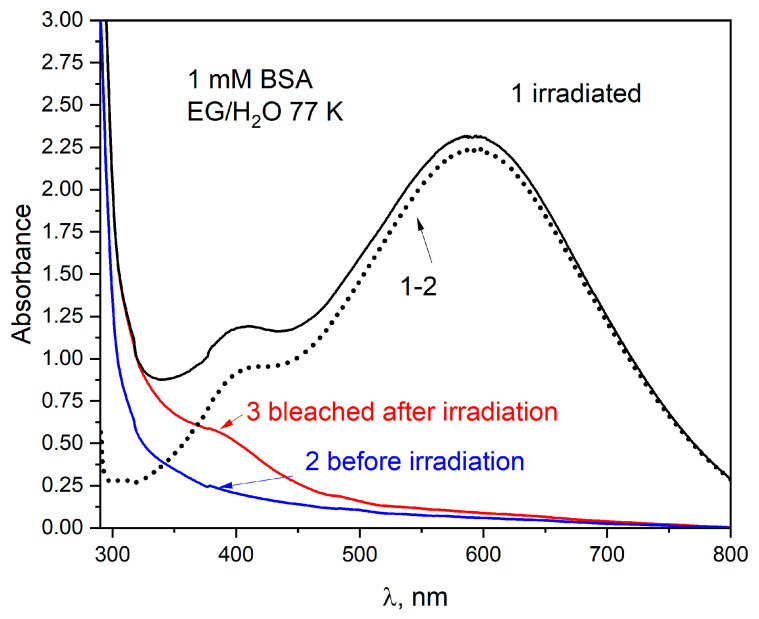
Absorption spectra of the BSA sample (1 mM) before (2) and after irradiation (1) with dose 6700 Gy at 77 K. Spectrum 3 recorded after photobleaching with visible light. The ethylene glycol–water glassy matrix (1:1 *v/v*). Sample thickness 1 mm.

The literature describes that the hydrated electron may react by tunneling over long distances within biomolecules [32,33]. Experimental studies of electron tunneling in proteins were initiated by DeVault and Chance in 1966 [34]. In our research, we attempted to explain whether electrons enter BSA directly or via subsequent scavenging by disulfide bridges. Steady-state radiolysis using an electron beam was performed at low temperatures. The ethylene glycol–water glassy matrix, containing BSA, was irradiated at 77 K. Under these conditions, many primary species and their reactions can be investigated. The absorption spectra of the 1 mM BSA at 77 K were recorded before and after irradiation with a dose of 6700 Gy and are presented in Figure 10. After irradiation, the absorption spectrum of the glassy sample shows two bands with a maximum around 410 and 590 nm (curve 1). The band centered at 590 nm can be attributed to the trapped electrons. The absorption spectrum of irradiated glassy BSA after subtraction of absorption of the unirradiated sample is presented as a dotted line (Figure 10). Curve 3 represents the spectrum of BSA obtained after bleaching with the light of a sample. The optical bleaching was carried out with visible light. The bleaching causes the disappearance of the spectrum of trapped electrons. After removing the trapped electron spectrum, a band with a maximum around 400 nm is still present. In the same spectral range, a band for reduced HSA (product of electron attachment to disulfide bonds) was observed in pulse radiolysis measurements. For this reason, we assume that disulfide radical anions (CyS-SCy^•−^) derived from the electron attachment are probably responsible for the absorption spectrum with a maximum at around 400 nm at 77 K. It appears that electrons can reduce disulfide bridges which are in the BSA interior both by diffusion (in the case of a hydrated electron in an aqueous sample) or tunneling through the BSA at matrices at 77 K.

As a result of measurements of the scavenging electrons by CyS-SCy bridges inside albumins (HSA or BSA), we believe that their transfer is possible in the tunneling process. This is indicated by low-temperature radiolysis measurements (77 K) of the EG/H_2_O matrix containing HSA or BSA. In a matrix irradiated at liquid nitrogen temperature, electron diffusion is completely inhibited. However, the formation of reduced CyS-SCy bridges is observed (Figure 10 shows a pronounced shoulder around 400 nm). The electron can also react in its presolvated state (“dry” electron). For the albumin concentrations used (about 1 mM) in the experiments, dry electron capture does not play a significant role. This was also confirmed by pulse radiolysis measurements of liquid solutions used in low-temperature measurements.

**Figure 11:**
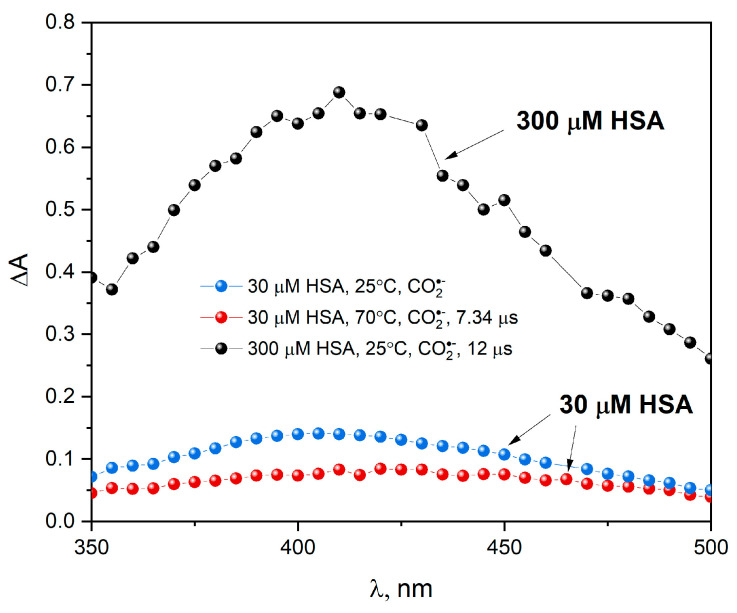
Transient absorption spectra of N_2_O-saturated buffer solution containing HSA (30 or 300 µM) and NaCOOH (0.1 M), obtained for irradiation dose 220 Gy, at 25 or 70 °C.

In the next stage of our studies, an HSA solution (30 or 300 µM) containing 0.1 M NaCOOH was studied using the pulse radiolysis technique and the absorption spectrum was recorded. The results from pulse radiolysis experiments show that ${CO}_{2}^{•-}$ radical anions react relatively fast (see comment below) and selectively with CyS-SCy groups (Figure 11). After electron pulse irradiation of the HSA solution, a new transient absorption peak with a maximum at 420 nm is formed (the redox potential of the ${CO}_{2}^{•-}$ radical anion is −2.0 V). BSA and HSA possess homologous amino acid sequences [35], resulting in almost identical secondary and tertiary structures. Results for the reaction of hydrated electrons or ${CO}_{2}^{•-}$ with both albumins are the same (almost the same rate constants, transient absorption spectra and aggregation process). When $N_{3}^{•}$ radicals react with HSA, similar transient absorption spectra were obtained, as in the case of the reaction of azide radicals with BSA, but their intensity is different [16]. For BSA, the absorption band is about twice as intense as the tryptophan radical in HSA. The higher absorbance in the transition spectra for BSA after one-electron oxidation is due to the presence of two tryptophan residues in the BSA structure, as opposed to one tryptophan residue in HSA. These observations confirm our previous measurements [1] that tryptophan residues in albumin are protected from attack by reducing agents.

**Figure 12:**
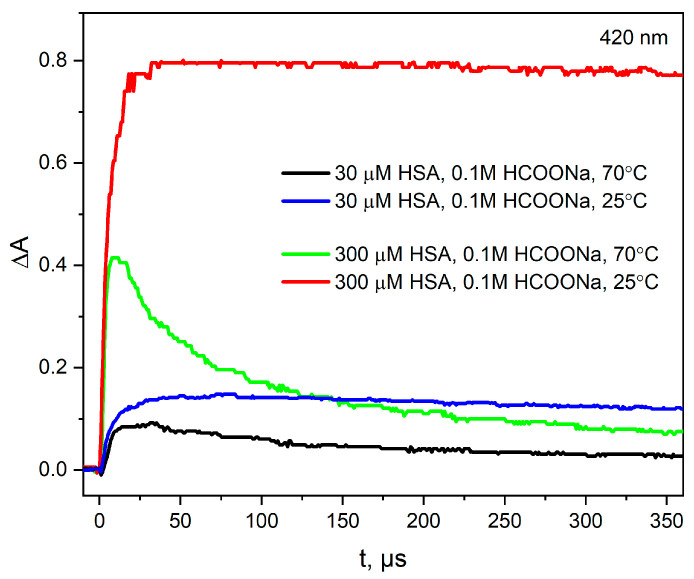
Time profiles of the absorbance recorded at 420 nm after pulse irradiation with a dose of 220 Gy of the N_2_O-saturated aqueous solution containing HSA (30 or 300 μM) and NaCOOH (0.1 M) at 25 or 70 °C.

Temperature measurements (Figure 12) of pulse radiolysis show that heating the HSA solution to 70 °C does not support the process of albumin reduction by ${CO}_{2}^{•-}$ anion radicals (the absorbance with a maximum at 420 nm decreases 1.75 times after heating the HSA solution compared to the sample irradiated at room temperature).

The value of the rate constant for the reaction of ${CO}_{2}^{•-}$ radical anions with HSA in buffer solution is around 5.7·10^8^ dm^3^ mol^−1^ s^−1^, in agreement with the value published in the literature of 7.0·10^8^ dm^3^ mol^−1^ s^−1^ (pH = 7.6) [34]. We are aware that this value is only an estimate. When analyzing the reactions of ${CO}_{2}^{•-}$ radical anions with a protein, the disproportionation reaction of these radicals should be taken into account, the rate constant of which is 6.5·10^8^ dm^3^ mol^−1^ s^−1^ at pH 7.0 [36].

While examining the formation of albumin aggregates induced by reactions with $e_{aq}^{-}$ or ${CO}_{2}^{•-}$ radical anions, we observed the formation of gels several times. These hydrogels are two-component systems consisting of a three-dimensional network of polypeptides chains and water that is trapped between macromolecules. Currently, hydrogels are used, among other applications, as dressings, supersorbents in the treatment of obesity, drug delivery systems, contact lenses and artificial cartilages. Since the pioneering work of Wichterle and Lim [37], ionizing radiation has been used to create hydrogels, which has resulted in the commercial production of many products [38].

Our observed ability to easily produce albumin hydrogels using radiation opens the way to preparing materials for therapeutic purposes. This is a very important issue to which we intend to devote our next publication. Key methods for irradiating human or bovine albumin solutions to obtain hydrogels will be presented below. The use of an electron accelerator is more effective than a gamma radiation source. To obtain gels using the radiation method, the concentrations of albumin solutions must be high, greater than or equal to 300 µM. Both small and large doses of ionizing radiation can be used to effectively produce hydrogels. Small doses require heating the irradiated solutions to a high temperature of 70 °C. When ${CO}_{2}^{•-}$ radical anions were used to reduce albumin, even with a few electron pulses at a dose of 820 Gy in HSA solution (300 µM, 70 °C), a gel was generated. When the hydrated electron was used to initiate the HSA aggregation process (under the conditions described above for ${CO}_{2}^{•-}$ radical anions), a gel was also obtained for only a slightly higher dose. This may be due to the fact that sodium formate changes the structure of HSA to some extent and facilitates the reduction of CyS-SCy bridges. For practical reasons, it is essential that both sodium formate and *t-*BuOH are non-toxic and suitable for use in cosmetics [39,40]. We also obtained hydrogels at room temperature using approximately 10 times higher doses compared to measurements at 70 °C. Irradiation of HSA solutions at room temperature is more promising in obtaining hydrogels. The key to the effective preparation of hydrogels is the method of delivering the electron beam to the starting solutions. The time interval between electron pulses from LINAC should correspond to the decay time of the reduced CyS-SCy^•−^ bridges in HSA, which, as we have shown, is several seconds [1]. In our measurements, we used a pulse repetition rate of approximately 0.5 Hz. To develop a procedure for obtaining commercial gels, this parameter must be optimized. The rate of energy delivery of ionizing radiation to albumin solutions under reducing conditions affects the rheological properties of the gels and their degree of transparency. In some experiments we managed to obtain clear, transparent gels; in others, they were yellow and opalescent. We presented the absorption spectrum of the clear gel in Figure S2 in the Supplementary Materials. The emission spectra of the hydrogel differ from those of the spontaneous aggregates, as shown in Figure S2. At the same time, the emission spectra of hydrogels are very similar to analogous spectra of aggregates obtained as a result of irradiation of solutions in conditions that do not lead to gelation. This observation suggests that intermolecular disulfide bridges are formed both in hydrogels and in aggregates obtained as a result of irradiation of HSA solutions (subsequent electron pulses are delivered every several dozen seconds or later).

Measurements of the decay times of light emission generated in albumin solutions using short laser pulses provide helpful information on the spontaneous and ionizing radiation-induced aggregation of HSA or BSA [17]. This technique should be used with great caution for complex systems such as protein solutions. Differences in the emission lifetime of aggregates depend on the initial concentrations of albumin solutions, pH, measurement temperature and hysteresis of temperature changes. Valuable information was provided from the analysis of the emission time measurements (after excitation with a 337 nm pulse in room temperature for 420 nm) of the solutions: a) the initial BSA solution (300 µM, 0.1 M t-BuOH), b) solution “a” after irradiation at 70 °C with a dose of 1300 Gy (30 pulses every 10 s) and c) the solution that absorbed the same total dose, but with high repeatability (1300 Gy; 30 pulses in 15 s). The BSA aggregates induced by ionizing radiation are characterized by specific fluorescence spectra compared to the emission of non-irradiated solutions (Figure S3 in Supplementary Materials). The excitation of BSA solutions with the laser light with a wavelength of 337 nm allows registration of the emission of excited protein aggregates only because this light is not absorbed by the monomeric form of BSA. The emission decay at 420 nm is characterized by a two-exponential function with an average time of 2.29 ns (t_1_ = 0.49 ns; f_1_ = 0.51; t_2_ = 4.16 ns; f_1_ = 0.49) for neat solution and 3.21 ns (t_1_ = 0.52 ns; f_1_ = 0.36; t_2_ = 4.75 ns; f_1_ = 0.64) for both the irradiated solution and the hydrogel. It can therefore be postulated that the properties of albumin aggregates generated by hydrated electrons are the same in solution and in hydrogel.

### 2.2. Raman Spectroscopy

**Figure 13:**
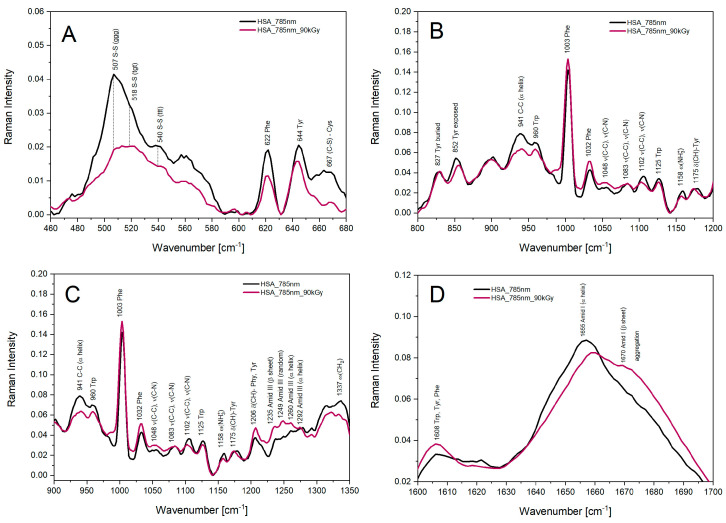
The Raman spectra of HSA (300 μM) samples containing *t-*BuOH (0.1 M) in the 680–460 cm^−1^ region (**A**); in the 1200–800 cm^−1^ region (**B**); in the 1350–900 cm^−1^ region (**C**); and in the 1700–1600 cm^−1^ region (**D**) before (black line) and after irradiation (pink line) with the dose 90 kGy. The excitation wavelength was 785 nm.

To study the reaction of $e_{aq}^{-}$ with HSA, we applied Raman spectroscopy. The changes in the secondary structure of a protein can be analyzed using this technique. Importantly, Raman spectroscopy is highly sensitive to the symmetrical vibrational modes of aromatic side chains, i.e., phenylalanine, tyrosine and tryptophan. In particular, the analysis of the S–S stretching region suggested the radical species caused modifications in the 17 disulfide bridges of HSA [22]. Figure S4 (in Supplementary Materials) shows Raman spectra recorded before and after γ-irradiation of N_2_-saturated aqueous solution containing 300 μM HSA and 0.1 M *t-*BuOH (90 kGy). The Raman spectrum of albumin consists of bands located in different spectral ranges depending on the frequency of vibration of the individual components of the sample. Disulfide bonds are assigned bands in the spectral range 550–500 cm^–1^ [41]. The strong Raman band in the spectral range 507–512 cm^–1^ corresponds to the S–S bond with the *ggg* geometry, which dominates mainly in the Cys–SS–Cys residues, while the less intense bands in the range 540–536 and 518–522 cm^−1^ indicate disulfide bonds occurring in the CC-SS-CC moiety, with geometries *tgt* and *ggt*, respectively. After irradiation of the albumin sample, the intensity of the bands at 507 cm^–1^, 518 cm^–1^ and 540 cm^–1^ decreases, indicating the breaking of sulfur bonds or their modification (Figure 13A). In the HSA spectrum, bands with low intensity in the spectral range 600–500 cm^–1^ can also be distinguished, which can be attributed to aromatic compounds. The band at 667 cm^–1^ corresponds to stretching vibrations in the Cys molecule. The intensity of this band also decreases after irradiation of albumin with a dose of 90 kGy. The Raman bands at 852 and 827 cm^–1^ observed in albumin before and after irradiation can be attributed to the Tyr molecule, buried deep in the albumin structure and exposed to the aqueous phase, respectively. After irradiation, the intensity of these bands practically does not change (Figure 13B). The bands in the region 800–1200 cm^−1^ correspond to C-C and C-N bonds in aliphatic amino acid molecules. Slight changes in the intensity values of these bands after irradiation of the studied sample indicate that the aliphatic groups are insignificantly modified after γ-irradiation. The amide bands I and III, used to study the secondary structure of proteins, have the following values: (1) amide I: 1645–1655 cm^−1^ (α helix), 1670–1680 cm^−1^ (β structure), 1660–1670 cm^−1^ (disordered structure); and (2) amide III: 1270–1280 cm^−1^ (α-helix), 1230–1240 cm^−1^ (β structure), 1240–1260 cm^−1^ (disordered structure). The effect of radiation on the HSA secondary structure is pronounced (Figure 13C). Ionizing radiation causes a decrease in the intensity of the 930–950 cm^–1^ and 1330–1340 cm^–1^ bands, indicating the loss of the α-helix structure. On the other hand, the increase in the intensity of the bands in spectral region 1235–1250 cm^–1^ indicates the predominance of the β sheet structure of the albumin after irradiation. The increase in the intensity on the band arm at 1670 cm^−1^ is due to the formation of protein aggregates, i.e., antiparallel β sheet structures (Figure 13D). Analogous changes in the Raman spectrum of HSA after irradiation with a dose of 50 and 300 Gy have been described in the literature [42,43].

The analysis of Raman spectra of HSA confirmed that as a result of the γ-irradiation of the albumin sample under reducing conditions, changes in the native protein structure occur and lead to the aggregation of albumin molecules in the buffer solution and thus to the formation of antiparallel intermolecular β sheet structures. Significant changes in the three-dimensional structure of human albumin may be the result of changes in the polypeptide backbone and the breaking of disulfide bonds in the HSA structure caused by irradiation of the sample. The conducted experiments confirmed that hydrated electrons were directly scavenged by disulfide bridges within the protein structure.

### 2.3. Reversed-Phase High-Performance Liquid Chromatography Measurements

**Figure 14:**
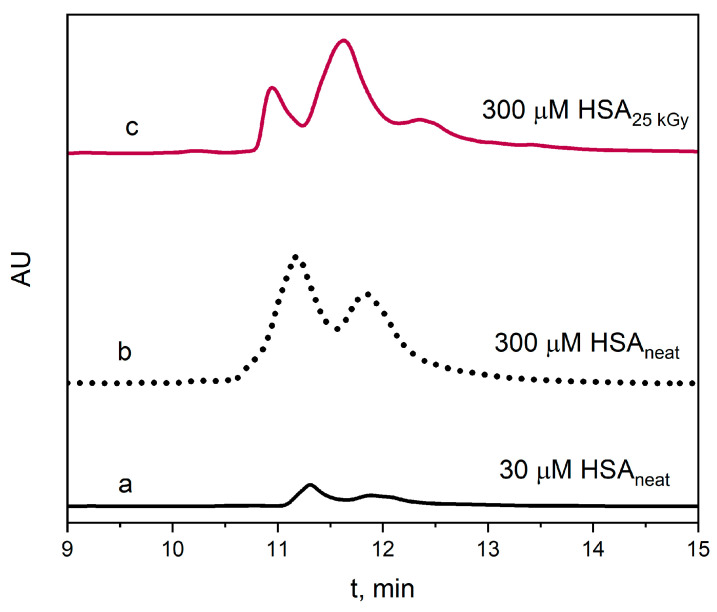
Chromatograms of (a) neat HSA solution (30 µM) before irradiation, (b) neat HSA solution (300 µM) before irradiation and (c) HSA solution (300 µM) after irradiation (25 kGy). The detection wavelength was 280 nm.

The reversed-phase high-performance liquid chromatography measurements were performed to confirm the presence of high-molecular-weight HSA aggregates generated in pulse radiolysis measurements. The vacuum-deaerated HSA solutions (30 or 300 μM) containing *t–*BuOH (0.1 M) were irradiated with an electron beam at a dose of 25 kGy and analyzed by RP-HPLC. The chromatograms obtained for the studied samples are presented in Figure 14. Based on the position of the peaks on the chromatogram and their peak areas before and after irradiation of HSA solutions, it can be concluded that the first peak with t_R_ =11.31 min characterizes the native form of HSA (the monomer form of albumin) and the second peak with t_R_ =11.83 min corresponds to HSA aggregates. The presence of a third peak with t_R_ =12.35 min in the chromatograms probably indicates the presence of high-molecular-weight HSA aggregates. Our measurements are consistent with the literature [44]. Our HPLC measurements clearly indicate that as the protein concentration increased or the HSA solution was exposed to ionizing radiation, the number of protein aggregates increased compared to the 30 µM HSA solution, in which the number of self-aggregates was the lowest. The ratio of the peak areas for the aggregates and the neat albumin form was the biggest for the albumin solution after irradiation with a dose of 25 kGy.

Furthermore, solutions of HSA were examined using HPLC analysis with the addition of Thioflavin T (ThT) (t_R_ = 8.69 min, λ_max_ = 415 nm), a dye commonly used to diagnose amyloid fibrils, both in vivo and in vitro. Previously, a preference for binding of molecular probes to aggregated forms of HSA as opposed to interaction with the monomeric form of HSA was observed [45]. The authors estimated on the base of HPLC measurements that in the HSA solution containing 1,3-di(1-pyrenyl)propane, 98% of the probe molecules were associated with protein aggregates (mainly HSA dimers) and not with HSA monomers.

ThT was added to the neat albumin solutions and solutions after irradiation. The quantum yield of ThT fluorescence is low and amounts to 0.001 (λ_exc_ = 350 nm). However, for the thioflavin–amyloid complex, the fluorescence is tens of times more intense. In the presence of protein aggregates, it was observed that the absorbance of thioflavin increased at the maximum of the ThT band (λ_max_ =415 nm). In addition, the maximum of the absorption band of ThT is red-shifted (415 nm → 420 nm) in the presence of aggregates compared to the buffer solution of the dye. The results from the spectroscopic measurements are in good agreement with the HPLC analysis. The ThT peak area is the largest in the 300 µM HSA solution exposed to ionizing radiation and has the lowest value in the buffer solution of the dye (Table 1).

This confirms that thioflavin interacts with HSA aggregates and can be used as a marker of aggregated forms of albumin. In our upcoming publication, we intend to address important aspects of the interaction of ThT with human serum albumin in more detail.

## 3. Discussion

In this section, we will summarize our electron scavenging measurements and supplement them with literature data regarding the identification of reaction products of hydrated electrons with HSA or BSA.We can describe this process as follows. We have shown that the electron capture process is diffusion-controlled. The activation energy of electron scavenging is the same as the activation energy of self-diffusion of water. The rate constant for the reaction of hydrated electrons with albumin, HSA or BSA is almost the same and is equal to 1.1·10^10^ dm^3^ mol^−1^ s^−1^ for HSA and 1.3·10^10^ dm^3^ mol^−1^ s^−1^ for BSA. Half of the amount of $e_{aq}^{-}$ generated in the accelerator pulse reacts with CyS-SCy bridges inside the albumin structure. The contribution of dry electron capture can practically be neglected (the maximum contribution for the highest protein concentrations does not exceed 2% of the hydrated electron concentration in the initial solution without albumin). Our Raman spectroscopy studies indicate strong modification of disulfide bonds and confirm aggregation of albumins as well.

Low-temperature measurements indicate the possibility of electron tunneling by the protein shell of HSA to specific CyS-SCy bridges. Although the possibility of tunnelling electron transfer was proposed even for liquid systems, this concerned very specific objects such as ferritin [32,46]. When studying electron transfer tunneling in low-temperature matrices using pulsed radiolysis, very high concentrations of scavengers are usually used, even in the order of mol dm^−3^. This is due to the short electron tunneling distance of several dozen Å. The solubility of albumin in aqueous solutions is limited and concentrations reach only 1–1.5 mM. The distance of the trapped electron from the albumin can be reduced by increasing the electron concentration. Our LINAC allows us to achieve a high electron concentration of two hundred and tens of μM, but this is far too low to observe tunnelling for the scavenger at a concentration of 1 mM. The trapped electron-scavenger distance can be reduced in the matrix, generating a very high electron concentration. We previously used this strategy for berberine in a glassy sample (H_2_O/EG 1/1 *v/v*) at a dose of 35.2 kGy at 77 K. The yield of trapped electrons in aqueous ethylene glycol glasses at 77 K is approximately 1.5/100 eV (0.15 μM/J) [47]. For a dose of 35.2 kGy, the concentration of the trapped electron is higher than 5 mM and is sufficient for the electron to react with the scavenger. For albumin, we observed electron capture for lower combined trapped electron and BSA concentrations of approximately 2 mM (1 mM scavenger and 1 mM trapped electron). This indicates a very high efficiency of albumin in the scavenging of trapped electrons. It should be noted that the albumin molecule is different from the classical scavenger molecule. The process of electron capture by a protein is more complex than the classic reaction of the electron with a scavenger molecule. The clear difference is due to the much larger dimensions (80 × 80 × 30 Å) of the protein compared to classical scavenger molecules (radius of order a few Å). Additionally, the albumin molecule can be considered a super scavenger due to the presence of many residues in its structure that are potential scavengers, including CyS-SCy bridges. Analyzing the formation of reduced CyS-SCy bridges in pulse radiolysis measurements, it can be formally assumed that their concentration is 17 times higher than the concentration of albumin itself. The low efficiency of reduced CyS-SCy formation was observed at room temperature (half of the amount of $e_{aq}^{-}$ produced in the accelerator pulse reacts with CyS-SCy bridges). This indicates that in a neutral pH solution, most of the disulfide bridges are buried inside the albumin and are not exposed to the water phase.

The results of our pulsed radiolysis measurements indicate that the electron diffuses into the protein. This is evidenced by the consistency of the kinetics of decay of hydrated electrons with the kinetics of formation of reduced disulfide bridges. The results of the reduction of HSA or BSA as a result of the reaction with ${CO}_{2}^{•-}$ indicate that the reducing agent penetrates deep into the albumin structure. The rate of the electron transfer from ${CO}_{2}^{•-}$ anion radicals to the disulfide groups in albumin was found to be 21 times slower than the rate of electron scavenging. This fact is due to the lower reducing capacity of ${CO}_{2}^{•-}$ anion radicals and the increased difficulty of its penetration into disulfide residues, which is a consequence of the relatively large size of ${CO}_{2}^{•-}$ anion radical particles. Interestingly, the efficiency of CyS-SCy^•−^ radical formation is high, comparable to that observed for the reaction with an electron. This indicates that those CyS-SCy residues that are easily accessible to the electron or ${CO}_{2}^{•-}$ anion radicals are reduced. Because of the lower redox potential, ${CO}_{2}^{•-}$ anion radicals react more selectively than $e_{aq}^{-}$ with albumin. We observed the same effectiveness of creating CyS-SCy^•−^ radicals as a result of the slow reaction of albumin with ${CO}_{2}^{•-}$ anion radicals and as a result of the rapid reaction of $e_{aq}^{-}$ with proteins.

It has long been known that within polypeptides, enzymes or proteins exposed to ionizing radiation, long-range electron transfer from tyrosine residues to semi-oxidized tryptophan moiety occurs [48,49]. The distance over which electron transfer was observed is not very large and is close to 15 Å. This is a consequence of the low value of the reaction driving force (small difference in redox potentials of Trp^•^ and Tyr). The discussed electron transfer process occurs on a long time scale, of the order of hundreds of microseconds. The slow, long-range electron transfer rate constant of 0.2 s^−1^ determined is independent of HSA concentration and radiation dose, consistent with an intramolecular process. This is the slowest rate constant so far reported for an intramolecular electron transfer [50].

During pulse radiolysis of albumin solution under reductive stress conditions, the mechanism involving the migration of electrons from the protein surface to the interior of the protein molecule may not need to be utilized. However, this does not imply that we reject the model in which electrons initially attach to the protein surface and then rapidly migrate to the protein, ultimately settling into disulfide moieties [51]. To observe this electron transfer, a pulse radiolysis system with absorption detection in the picosecond domain or shorter is required.

## 4. Materials and Methods

### 4.1. The Sample Preparation

Essentially fatty acid-free albumin from human serum (HSA, A1887) and bovine serum albumin (BSA, A7030) were obtained from Sigma-Aldrich (St. Louis, MO, USA)/Merck (Darmstadt, Germany) and used as received. Protein solutions were prepared in ultrapure water in 10 mM phosphate buffer (PBS, pH 7.2). Water was purified with the Hydrolab SPRING 20UV system. HSA or BSA was dissolved in phosphate buffer solution to appropriate concentrations immediately before measurements. The final concentrations of the solutions were verified spectrophotometrically (ε_280 nm_ = 35,500 M^−1^ cm^−1^ for HSA, ε_280 nm_ = 43,800 M^−1^ cm^−1^ for BSA).

### 4.2. Optical Measurement

Steady-state emission spectra of the studied albumin solution were carried out using the Aminco-Bowman Series 2 (Hamamatsu R928, Hamamatsu Photonics, Shizuoka, Japan) and absorption spectra were recorded using a Perkin Elmer Lambda 750 spectrophotometer. Optical measurements are described in detail elsewhere [16].

### 4.3. Pulse Radiolysis

Pulse radiolysis measurements were performed using a 6 MeV linear accelerator (LINAC) ELU–6E operating in single-pulse mode. The methodology and equipment for pulse radiolysis study with optical detection have been described in [16]. Depending on the needs, the solutions were saturated with ultrapure nitrogen, nitrous oxide or oxygen or were deaerated under vacuum. The samples subjected to electron beam irradiation also contained 0.1 M *tert*-butanol (*t-*BuOH) as a scavenger of ^•^OH radicals. The molar absorption coefficient of reduced HSA (ε for CyS-SCy^•−^) is below 10^4^ dm^3^ mol^−1^ s^−1^ [52,53].

Pulse radiolysis has been utilized to investigate the electron scavenging reaction by BSA at 77 K. The methodology for low-temperature pulse radiolysis measurements has been detailed in the literature [54]. In the case of low-temperature measurements, solutions were prepared immediately before freezing the sample. The holder with the brass cuvette was immersed in liquid nitrogen, and then, after freezing, the studied solution was injected into the cuvette and the holder was immersed in liquid nitrogen again. After freezing and obtaining the glass of the solution, the holder with the cuvette is mounted in a cryostat. The ethylene glycol–water glassy matrix was examined. Bovine serum albumin (BSA) was used as an electron scavenger. The glassy sample was irradiated at 77 K in a Optistat DN cryostat (Oxford Instruments, OINS, Tubney Woods, Abingdon, Oxon, OX13 5QX, UK). Absorption spectra of the frozen sample of BSA were recorded using a Perkin Elmer Lambda 750 UV-VIS spectrophotometer.

### 4.4. Reversed-Phase High-Performance Liquid Chromatography Measurements

The reversed-phase high-performance liquid chromatography (RP-HPLC) analysis was performed using a Waters HPLC system consisting of a diode array detector (Waters 2998), a binary HPLC pump (Waters 2545) and an autosampler (Waters 2767). The separation of the monomer from the HSA aggregates was carried out on a chromatographic column XBridge C18 OBD (4.6 mm × 100 mm) with a particle size of 5 μm. The mobile phase flow rate was kept at 1 mL/min. The mobile phase A consisted of a 0.1% (*v/v*) aqueous solution of trifluoroacetic acid (TFA), and phase B consisted of a 0.1% (*v/v*) solution of trifluoroacetic acid in acetonitrile/water (70:30 *v/v*) under gradient elution conditions, as follows (Table 2).

The injection volume was 2 μL. The UV spectra were recorded over a range of 190–800 nm, and chromatograms were acquired at 280 nm (for HSA aggregates) and 415 nm (for Thioflavin T, ThT). ThT was dissolved in buffer or examined solutions of HSA (300 µM) to appropriate concentrations immediately before measurements. The final concentration of the ThT was 30 µM in all samples. The experiments were performed in triplicate at room temperature. The MassLynx software (version 4.1) was used for instrument control and data acquisition.

### 4.5. Raman Spectroscopy

Raman spectroscopy measurements were performed with a confocal Raman microscope (Alpha 300RSA, WITec, Ulm, Germany) equipped with 355, 532 and 785 nm diode lasers, a 300 mm triple grating imaging spectrometer (Acton SpectraPro SP–2300; Princeton Instruments Inc., Acton, MA, USA), a thermoelectrically cooled CCD camera (Andor, Ireland) and a 40× objective lens [55]. Raman peak positions were checked using a reference sample (a silicon wafer Raman peak at 520.7 cm^−1^). Raman spectra were collected using a 0.3 s integration time (785 nm, 80 mW).

## Figures and Tables

**Table 1:** The thioflavin peak area in HSA solutions before and after irradiation.

Λ = 415 nm	ThT_buffer_	300 µM HSA, 0 kGy	300 µM HSA, 25 kGy
Peak area (t_R_ = 8.69 min)	6970	8850	13,955

**Table 2:** The gradient elution conditions on a chromatographic column.

Time, min	0	12	19	43	44	54
%A	70	40	40	25	70	70
%B	30	60	60	75	30	30

## Data Availability

Data are contained within the article and Supplementary Materials.

## Supplementary Materials

The following supporting information can be downloaded at https://www.mdpi.com/article/10.3390/ijms25169009/s1.
