# Supplementary material for: Aggregation of Albumins under Reductive Radical Stress
Source: Int J Mol Sci. 2024 Aug 19;25(16):9009. doi: 10.3390/ijms25169009 (PMC11354859; doi:10.3390/ijms25169009)
Supplement: Supplementary file 1 [file ijms-25-09009-s001.zip › ijms-3117235-supplementary.pdf]

Supplementary Materials

Karolina Radomska, Liwia Lebelt, Marian Wolszczak*

* Correspondence: marian.wolszczak@p.lodz.pl

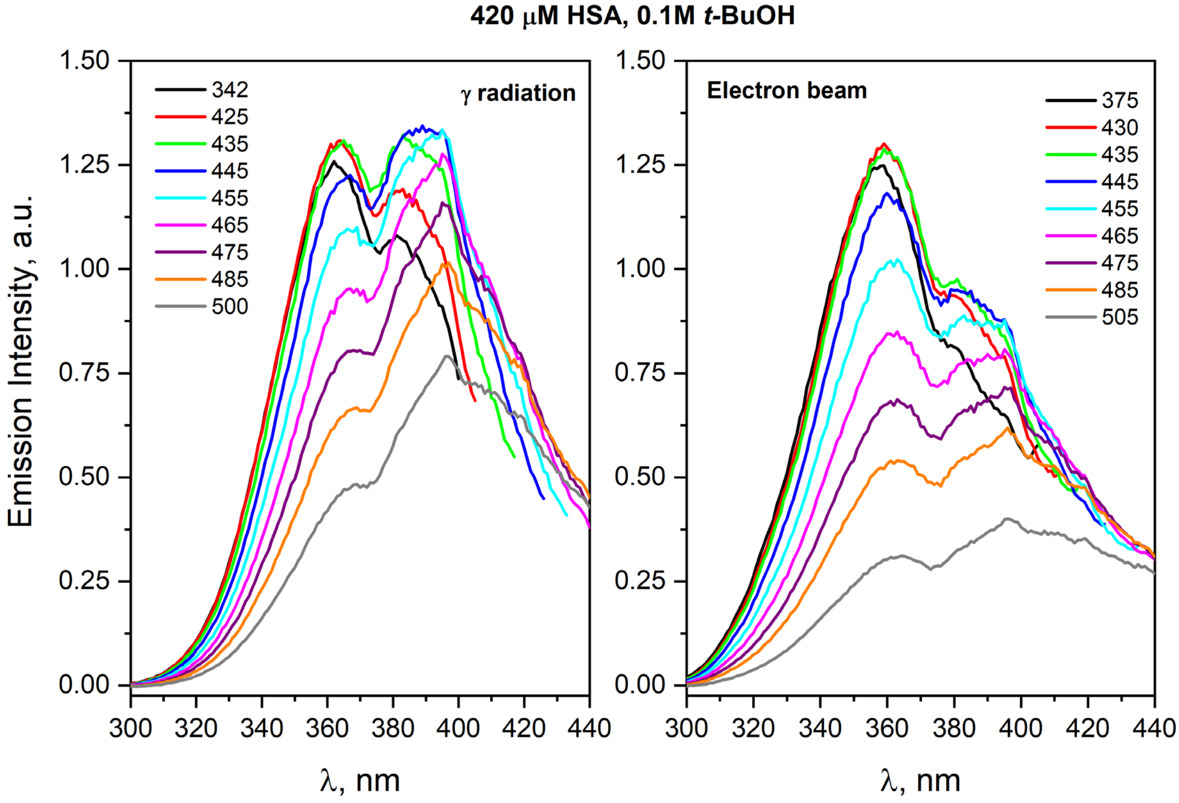

Figure S1. Emission excitation spectra of the N_2-saturated HSA solutions (420 μM) containing 0.1 M *t*-BuOH recorded after γ-irradiation (50 kGy) or after irradiation with electron pulses (90 kGy). The emission detection wavelengths are given in the figure.

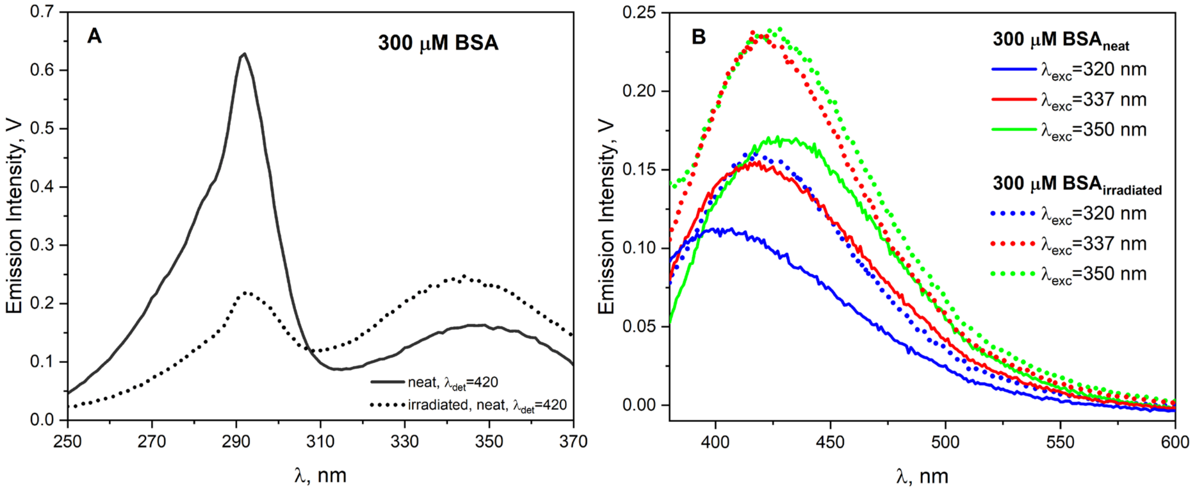

Figure S2. **A**. Emission excitation spectra of the neat BSA solution (300 μM) and solution of BSA (300 μM) obtained for irradiation dose 1300 Gy. The emission wavelengths were 420. **B.** Emission spectra of the neat and irradiated BSA solutions (300 μM, 1300 Gy). The excitation wavelengths were 320, 337 and 350 nm.

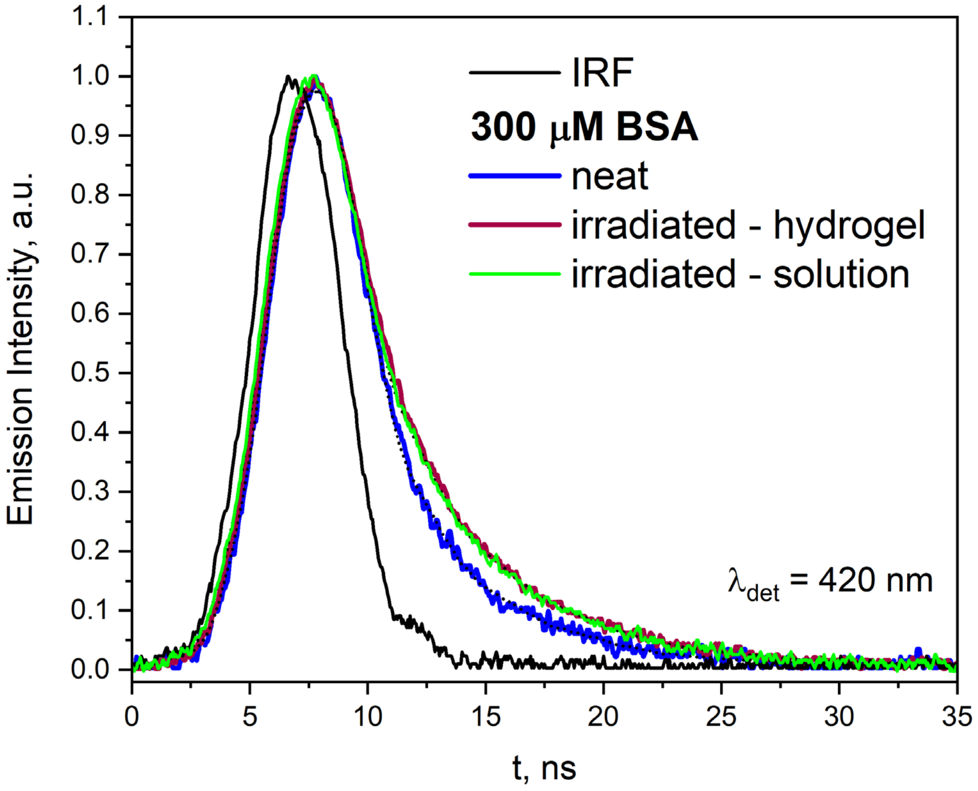

Figure S3. Decays of fluorescence of the neat BSA solution (300 μM) and solution of BSA (300 μM) obtained for irradiation dose 1300 Gy (decays for hydrogel and solution of BSA).

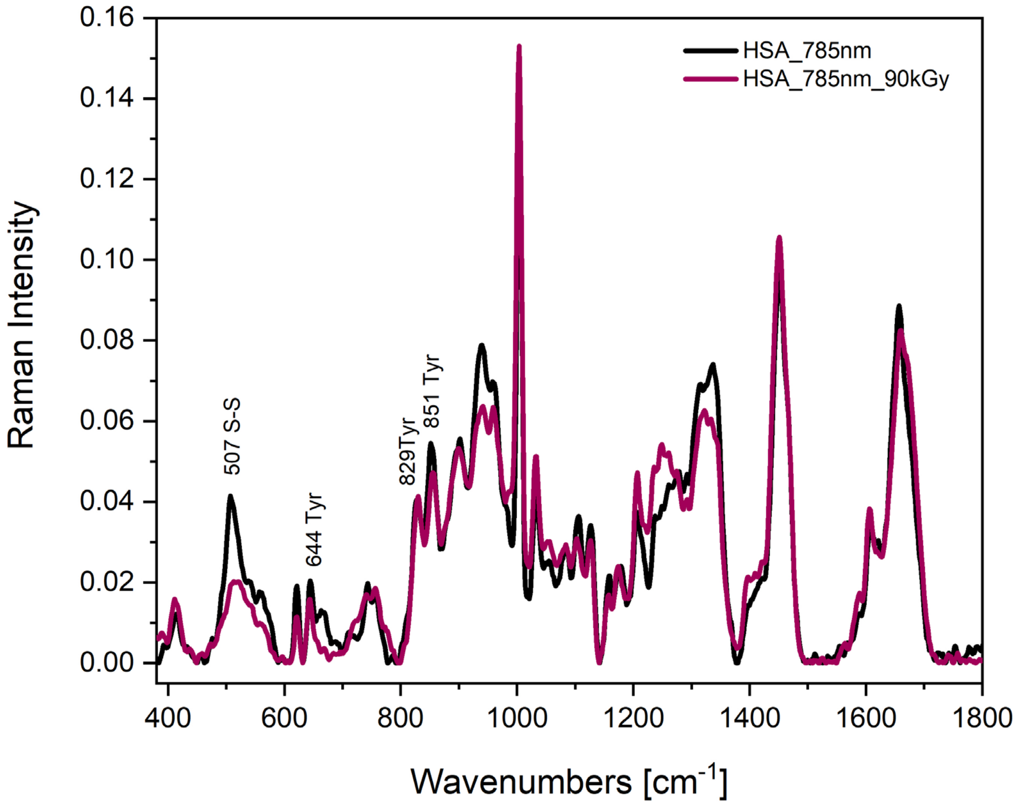

Figure S4. The Raman spectra of HSA (300 μM) samples containing *t*-BuOH (0.1 M) in the 1800–400 cm^{-1} region before (black line) and after irradiation (pink line) with the dose 90 kGy. The excitation wavelength was 785 nm.
